# Identification of somatic and germline mutations using whole exome sequencing of congenital acute lymphoblastic leukemia

**DOI:** 10.1186/1471-2407-13-55

**Published:** 2013-02-04

**Authors:** Vivian Y Chang, Giuseppe Basso, Kathleen M Sakamoto, Stanley F Nelson

**Affiliations:** 1 Department of Pediatrics, Division of Hematology-Oncology, University of California, Los Angeles, 10833 Le Conte Ave., MDCC A2-410, Los Angeles, CA, 90095, USA; 2 Woman and Child Health Department, University of Padova, Via Giustiniani, 335128, PADOVA, Italy; 3 Department of Pediatrics, Division of Hematology/Oncology, Stanford University School of Medicine, CCSR-1215C, 269 Campus Drive, Stanford, CA, 94305-5162, USA; 4 Department of Human Genetics, Pathology and Laboratory Medicine, and Psychiatry, University of California, Los Angeles, 695 Charles E. Young Drive South, Los Angeles, CA, 90095, USA

**Keywords:** Pediatric leukemia, Congenital acute lymphoblastic leukemia, Exome sequencing

## Abstract

**Background:**

Acute lymphoblastic leukemia (ALL) diagnosed within the first month of life is classified as congenital ALL and has a significantly worse outcome than ALL diagnosed in older children. This suggests that congenital ALL is a biologically different disease, and thus may be caused by a distinct set of mutations. To understand the somatic and germline mutations contributing to congenital ALL, the protein-coding regions in the genome were captured and whole-exome sequencing was employed for the identification of single-nucleotide variants and small insertion and deletions in the germlines as well as the primary tumors of four patients with congenital ALL.

**Methods:**

Exome sequencing was performed on Illumina GAIIx or HiSeq 2000 (Illumina, San Diego, California). Reads were aligned to the human reference genome and the Genome Analysis Toolkit was used for variant calling. An in-house developed Ensembl-based variant annotator was used to richly annotate each variant.

**Results:**

There were 1–3 somatic, protein-damaging mutations per ALL, including a novel mutation in Sonic Hedgehog. Additionally, there were many germline mutations in genes known to be associated with cancer predisposition, as well as genes involved in DNA repair.

**Conclusion:**

This study is the first to comprehensively characterize the germline and somatic mutational profile of all protein-coding genes patients with congenital ALL. These findings identify potentially important therapeutic targets, as well as insight into possible cancer predisposition genes.

## Background

Acute lymphoblastic leukemia (ALL) is the most common type of cancer diagnosed in children. Congenital ALL is a rare and aggressive subtype of ALL defined as diagnosis within the first month of life. A recent study of 30 patients with congenital ALL treated on the Interfant-99 protocol reported a 2-year event-free survival of 20% despite intensive chemotherapy [1]. This is significantly worse than the 5-year event-free survival of older children with ALL, which approaches 80% [2]. Although the 11q23 rearrangement is the most common cytogenetic abnormality in congenital and infant ALL [3], studies demonstrate that this rearrangement is not sufficient for leukemogenesis [4,5] and does not entirely explain the aggressiveness of ALL in this population of patients [6-8].

These data demonstrate that congenital ALL is a biologically different disease, and therefore may be caused by a distinct set of mutations in ALL blast cells that differ from blasts from older patients. Whole-exome sequencing can be used to characterize the majority of amino acid encoding base positions of the genome. When applied to cancer, this method can identify somatic mutations that may contribute to leukemogenesis, as well as germline mutations that may reveal cancer predisposition genes [9-12]. In this paper, we report whole-exome sequencing on four paired tumor-normal samples from patients with congenital ALL and fully characterize the germline and somatic mutations. In addition, healthy parents of one patient were also sequenced to verify any inherited germline mutations. Our results demonstrate that there are very few somatic mutations in cALL and that there are potential druggable targets that may provide new therapeutic options to improve outcomes.

## Methods

The UCLA Institutional Review Board approved this study, which was carried out in compliance with the Helsinki Declaration, and all participants, or parents of participants, provided written informed consent before samples were collected.

### Patient characteristics

We collected peripheral blood at diagnosis and remission bone marrow from four patients with congenital ALL (Table 1). The institutional review board reviewed and approved this study.

**Table 1:** Sample characteristics

**ID**	**Translocation**	**% peripheral blasts at diagnosis**
Sample 1	t(4;11)	92%
Sample 2	t(4;11)	94%
Sample 3	t(11;19)	80%
Sample 4	Negative	95%

### DNA extraction and sequencing

Tumor genomic DNA was extracted from peripheral blood at diagnosis and normal genomic DNA was extracted from remission bone marrow using QIAmp DNA Minikit (Qiagen, Valencia, California). Genomic DNA was enriched for coding exons using Sure Select Human All Exon for sample 1, and Human All Exon 50Mb kits for samples 2–4 (Agilent, Santa Clara, California). Sample 1 was sequenced on one full lane of the Illumina Genome Analyzer IIx as 76x76 base paired-end reads as well as one full lane of the HiSeq2000 as 50x50 base paired-end reads and reads were merged for downstream analysis (Illumina, San Diego, California). Leukemia sample numbers 2 through 4 and parents of sample 1 were sequenced on one full lane of the HiSeq2000 as 100x100 base pair, paired-end reads, while the germlines of samples 2–4 were sequenced on one full lane of the HiSeq2000 as 50x50 base pair, paired-end reads.

### Variant calling and filtration

Sequence reads were aligned to the human reference genome build 37, using Novoalign (novocraft.com). Post-processing of reads was performed using Samtools (samtools.sf.net) and Picard (picard.sf.net) for removal of PCR duplicates, merging, and indexing [13].

The Genome Analysis Toolkit (GATK) was used for recalibration of base quality, variant calling, filtration and evaluation [14,15]. Quality scores generated by the sequencer were recalibrated by analyzing the covariation among reported.

Quality score, position within the read, dinucleotide, and probability of a reference mismatch. Local realignment around small insertions and deletions (indels) was performed, using GATK's indel realigner to minimize the number of mismatching bases across all reads. Statistically significant non-reference variants, single nucleotide substitutions (SNS) and small indels were identified using the GATK UnifiedGenotyper. The GATK VariantAnnotator annotated each variant with various statistics, including allele balance, depth of coverage, strand balance, and multiple quality metrics. These statistics were then used in an adaptive error model to identify likely false positive SNSs, using the GATK VariantQualityScoreRealibrator (VQSR). Single nucleotide substitutions with a low VQSR score were filtered out, leaving a set of likely true variants. Hard filtering was applied to indels and only passing indels were used for subsequent analyses.

An in-house program based on the Ensembl database (http://www.ensembl.org) was used to further annotate variants with gene, transcript, and protein identifiers, conservation, tissue-specific expression, reference and alternate allele frequencies based on 1000Genomes (http://www.1000genomes.org/data), dbSNP132 (http://www.ncbi.nlm.nih.gov/projects/SNP), NHLBI (http://evs.gs.washington.edu/EVS) and NIEHS (http://evs.gs.washington.edu/niehsExome), among additional annotations.

### Germline analysis

Variants were filtered out if they were in non-coding regions, resulted in synonymous amino acid changes, or were predicted to have a benign change in protein function by Polyphen (http://genetics.bwh.harvard.edu/pph) or Sift (http://sift.jcvi.org). Variants were classified as rare if alternate allele frequencies were less than 1%.

Nonsynonymous, protein-damaging, and rare germline variants were intersected with known germline mutations that predispose to cancer syndromes, found in Cosmic [16]. Germline variants were also intersected with known DNA repair genes [17]. Germline variants in sample 1 were cross-checked with the parents’ sequence data to identify inherited versus de novo mutations. All germline and somatic variants at the last step of filtering were manually visualized using Integrated Genomics Viewer [18].

### Somatic analysis

Mutations were classified as somatic if they were rare and found in the tumor sample only with no evidence in the germline data. Fisher’s Exact test was performed on the reference and non-reference reads and p-value <1x10^-6^ was used as the cut-off for significance. Somatic mutations found in sample 1 were cross-checked with the parents’ sequence data to ensure they were indeed somatic and not alleles missed in the germline. Three somatic variants were excluded because they were present as non-reference reads in one or both parents.

### Polymerase chain reaction and capillary sequencing

The *SHH* mutation in Sample 1, *FLT3* mutation in Sample 3, and *DMBT*-*1* mutation in Sample 4 were validated using PCR and capillary sequencing. All primers for mutations were designed using Primer3Plus (http://www.bioinformatics.nl/cgi-bin/primer3plus/primer3plus.cgi) and ordered from Integrated DNA Technologies (Coralville, IA). Capillary sequencing was performed on Biosystems 3730 Capillary DNA Analyzer (Life Technologies, Carlsbad, CA). Raw and analyzed sequence results were visualized on Sequence Scanner v1.0 (Life Technologies, Carlsbad, CA). There was not sufficient DNA for Sample 2 to validate variants with PCR and capillary sequencing.

## Results

### Alignment and coverage statistics

The total number of reads per sample ranged from about 185,000,000-304,000,000 (Table 2). Sixty eight to ninety nine percent of reads aligned to the reference human genome and 87-94% of reads were covered at a minimum 20 times. The overall average coverage ranged from 107-210x.

**Table 2:** Alignment and coverage statistics by sample

	**Total Reads**	**Total Mapped**	**% Covered at 20x**	**Average Coverage**	**% PCR duplicates**
Sample 1 tumor	304,589,893	271,320,952	92%	210x	7.6
Sample 1 germline	295,105,503	263,056,333	90%	199x	8.9
Sample 1 mother	195,514,828	193,745,082	86%	147x	32.5
Sample 1 father	203,906,150	202,199,642	85%	145x	36.2
Sample 2 tumor	204,158,706	142,291,992	84%	141x	7.7
Sample 2 germline	243,212,434	220,803,923	82%	107x	14.5
Sample 3 tumor	185,947,244	127,115,774	83%	128x	7.6
Sample 3 germline	252,335,878	204,463,822	83%	111x	16.0
Sample 4 tumor	214,824,644	157,010,833	85%	149x	7.8
Sample 4 germline	239,034,182	215,730,577	83%	108x	15.7

**Figure 1:**
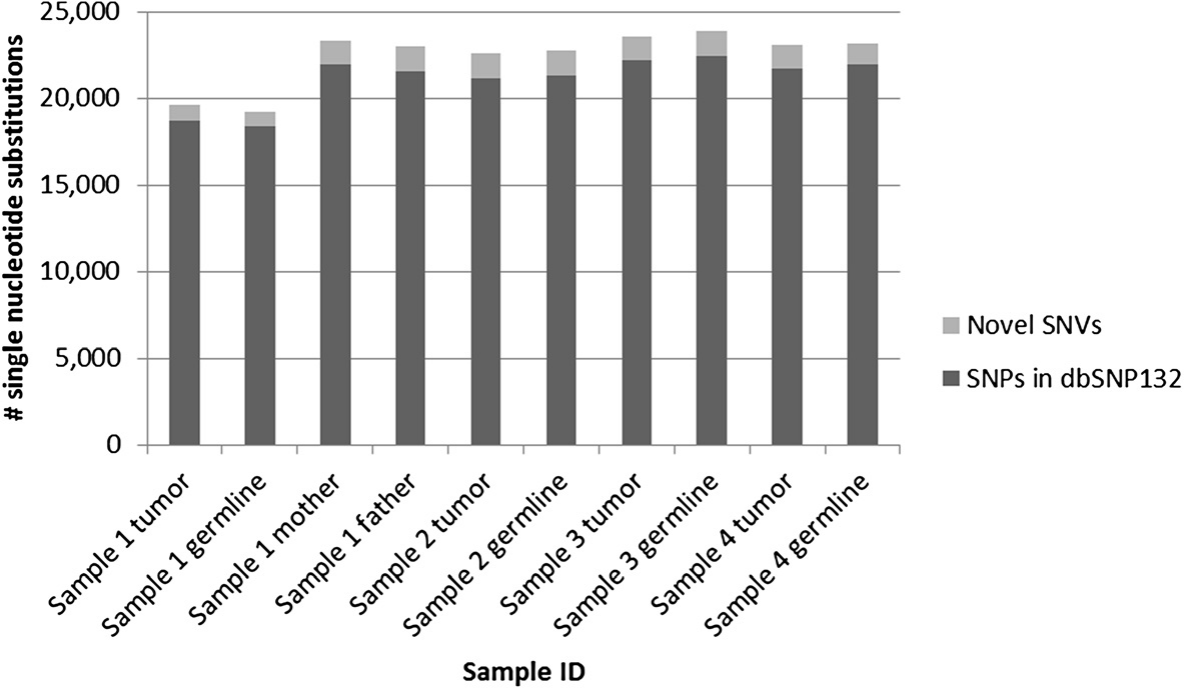
Number of single nucleotide substitutions per sample.

**Figure 2:**
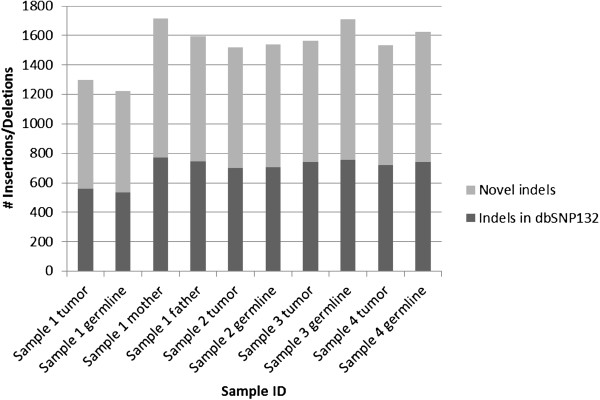
Number of small insertions and deletions by sample.

**Figure 3:**
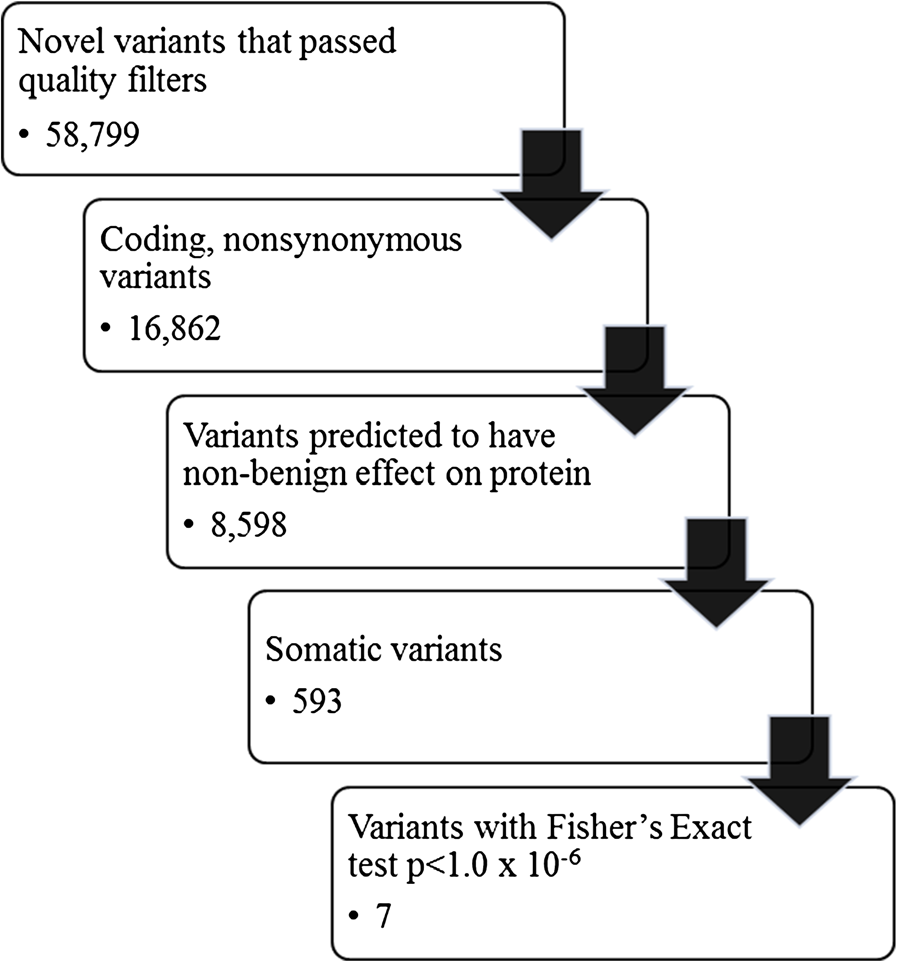
Number of variants by filtration step.

Each sample had 19,210-23,859 total single nucleotide substitutions. Greater than 93% of these were single nucleotide polymorphisms found in dbSNP132, with 99.8% concordance with the alternate allele found in dbSNP132. There were 791–1,462 novel single nucleotide variants per sample after removing polymorphisms found in dbSNP132 (Figure 1). Each sample had 1,222-1,716 total small indels. After removing polymorphisms found in dbSNP132, each sample had 688–943 novel indels (Figure 2). Variants were further prioritized if they were nonsynonymous, predicted to be damaging by either Sift or Polyphen, and rare in the general control population (Figure 3).

### Germline mutations

There were 2–6 germline mutations in each sample that were also in the Cosmic list of genes that have previously been associated with cancer predisposition [16]. Additionally, there were 7–13 germline mutations in each sample in genes that are known DNA repair genes. When comparing the congenital ALL samples with 28 control exomes from children without cancer sequenced in the same laboratory and analyzed with the same workflow, there were no statistically significant differences between mean numbers of mutations that overlapped with Cosmic germline genes or DNA repair genes (Table 3). Due to the small numbers of patients in each group, it was not possible to directly compare specific germline mutations within the cALL group and the control group.

**Table 3:** Comparison of mean overlap with Cosmic germline genes and DNA repair genes in patients with cALL and children without cancer

	**Mean overlap with Cosmic**	**Mean overlap with DNA repair genes**
4 cALL patients	5.18	9.50
28 control exomes	3.92	11.17
p-value	0.32	0.57

P-values calculated with Student’s t-test.

### Somatic mutations

There were 1–3 nonsynonymous, protein-damaging, rare variants found in each tumor sample with no evidence in the corresponding germline data using Fisher’s Exact p-value <1x10^-6^ on the reference and non-reference reads (Table 4). All these mutations were heterozygous. The two somatic mutations in sample 1 were homozygous reference in both parents.

**Table 4:** **Nonsynonymous**, **protein**-**damaging**, **rare somatic mutations with p**-**value** <**1x10**^-**6**^

**ID**	**Chrom**:**Pos**	**Gene**	**Amino acid change**	**Prediction(Sift, Polyphen)**
Sample 1	1:166039478	*FAM78B*	*/Y	.
	7:155599125	SHH*	G/S	Deleterious, Probably-damaging
Sample 2	13:28602340	*FLT3*	N/K	Deleterious, Probably-damaging
Sample 3	4:1388625-1388626	*CRIPAK*	.	.
	13:28592640	FLT3*	D/E	Deleterious, Probably-damaging
	X:140994655-140994657	*MAGEC1*	S/-	.
Sample 4	10:124380869	DMBT1*	H/N	Tolerated, Possibly-damaging

*Validated with PCR and capillary sequencing.

## Discussion

Although there has been significant progress in overall survival for children with ALL, newborns with congenital ALL continue to have poor prognoses despite intensive therapy. There is a need to identify new therapeutic targets in congenital ALL to rationally design treatment regimens that will produce sustained remissions with less toxicity. Additionally, understanding the molecular basis for congenital ALL may lead to novel insights into leukemogenesis and new cancer predisposition syndromes. This study is the first to comprehensively characterize the somatic and germline mutational profile of all protein-coding genes in four tumor-normal paired samples from patients born with congenital ALL.

Sample 1 had a somatic mutation in *SHH*, which has not previously been reported in ALL. The Hedgehog pathway is known to have a role in normal B-lymphocyte development and use of Hedgehog pathway inhibitors leads to decreased self-renewal potential [19]. The G143S mutation found in Sample 1 lies in a critical signaling region of the SHH protein that interacts with the SHH receptor, Patched (PTCH). Association of SHH with PTCH releases the inhibitory effect of PTCH on Smoothened (SMO), which allows for the propagation of SHH signals to activate transcription factors including GLI-1, 2, and 3 [20]. It is possible that this mutation has an activating effect on SHH that leads to dysregulation of downstream target genes.

Two of the four samples had somatic mutations in *FLT3*. Point mutations and internal tandem duplications in *FLT3* are known to be driver mutations in acute myelogenous leukemia (AML) but are also enriched in infant ALL [21]. Multiple oral FLT3 inhibitors have been tested in Phase 1 and 2 trials as single agents, as well as in combination with other chemotherapy agents for treatment of AML [22-25] with promising results. This study identified that single nucleotide substitutions in FLT3 are recurrent in ALL and infants with ALL might benefit from treatment with FLT3 inhibitors.

## Conclusion

This is the first study to perform exome sequencing on paired tumor and normal samples from patients with congenital ALL. Three of the four tumor samples had somatic mutations in genes that are druggable targets. Germline analyses did not reveal any clear set of cancer predisposition genes but a larger number of samples will need to be sequenced in order to delineate the role of DNA repair genes and known germline cancer predisposition genes, as well as to identify novel cancer predisposition genes.

As the cost of next-generation sequencing continues to decrease, patients and physicians will routinely encounter opportunities to supplement traditional morphology, flow cytometry, and cytogenetics tests with a base-pair level resolution of all variants in the exome as well as whole genome. High-throughput functional assays to validate the effect of all candidate driver mutations will be needed to fully take advantage of this level of mutational profiling. Additionally, inherited or de novo mutations in patients’ germlines will continue to expand currently known cancer predisposition syndromes and may eventually lead to approaches for earlier cancer detection and even cancer prevention.

## Competing interests

The authors declare no financial or non-financial competing interests.

## Authors’ contributions

VC carried out the sequence data analysis and drafted the manuscript. KS and SN participated in the design and coordination of the study, and helped to draft the manuscript. GB and KS recruited the patients. All authors read and approved the final manuscript.

## Authors’ information

Authors Kathleen M Sakamoto and Stanley F Nelson are both co-senior authors.

## Pre-publication history

The pre-publication history for this paper can be accessed here:

http://www.biomedcentral.com/1471-2407/13/55/prepub
